# Supplementary material for: Reversible induction of mitophagy by an optogenetic bimodular system
Source: Nat Commun. 2019 Apr 4;10:1533. doi: 10.1038/s41467-019-09487-1 (PMC6449392; doi:10.1038/s41467-019-09487-1)
Supplement: Supplementary file 1 — Supplementary Information [file 41467_2019_9487_MOESM1_ESM.pdf]

SUPPLEMENTARY INFORMATION

Reversible induction of mitophagy by an optogenetic bimodular system

Pasquale D'Acunzo et al.

Supplementary Figure 1

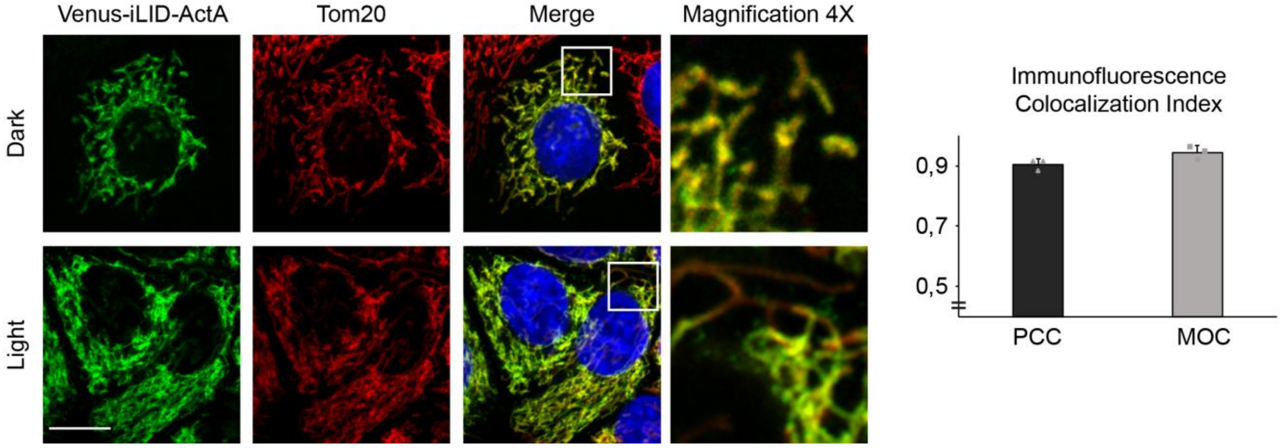

Supplementary Figure 1. *No signs of mitochondrial stress in Venus-iLID-ActA overexpressing cells, neither in the dark nor after 72h blue light stimulus.* Venus-iLID-ActA overexpressing HeLa cells were left in the dark or illuminated 72h with a pulsed (1s light, 1 min dark) blue light 7,2 W LED emitter. Subsequently, cells were fixed and stained for nuclei (DAPI 1 µg per ml, blue) and Tom20 (red). PCC and MOC of the green over the red signal of the Light condition were quantified in 10 random fields of 3 independent experiments. Inset: 4x magnification. Scale bar: 10 µm. Data shown: Mean ± S.E.M. Source data are provided as a Source Data file.

Supplementary Figure 2

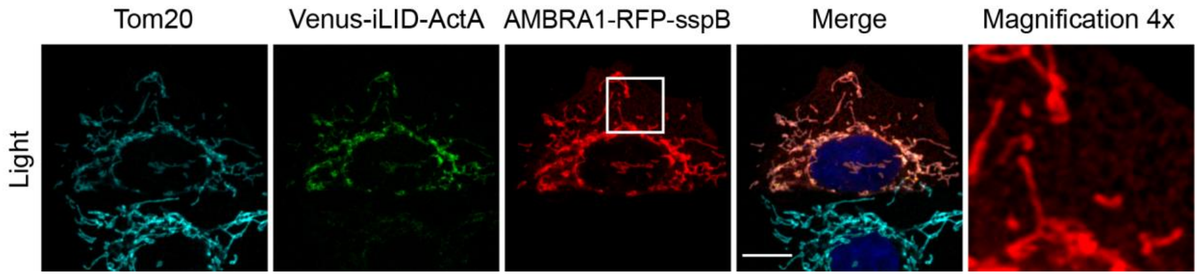

Supplementary Figure 2. *Upon blue light irradiation, few cells show cytosolic retention of the AMBRA1-RFP-sspB fusion protein, albeit very modest.* Representative immunofluorescence of a cell in which AMBRA1-RFP-sspB was partially retained in the cytosol (9,4±1,4% of all transfected cells in 3 independent experiments) when HeLa cells were irradiated and processed as indicated in Fig. 1c.

Supplementary Figure 3

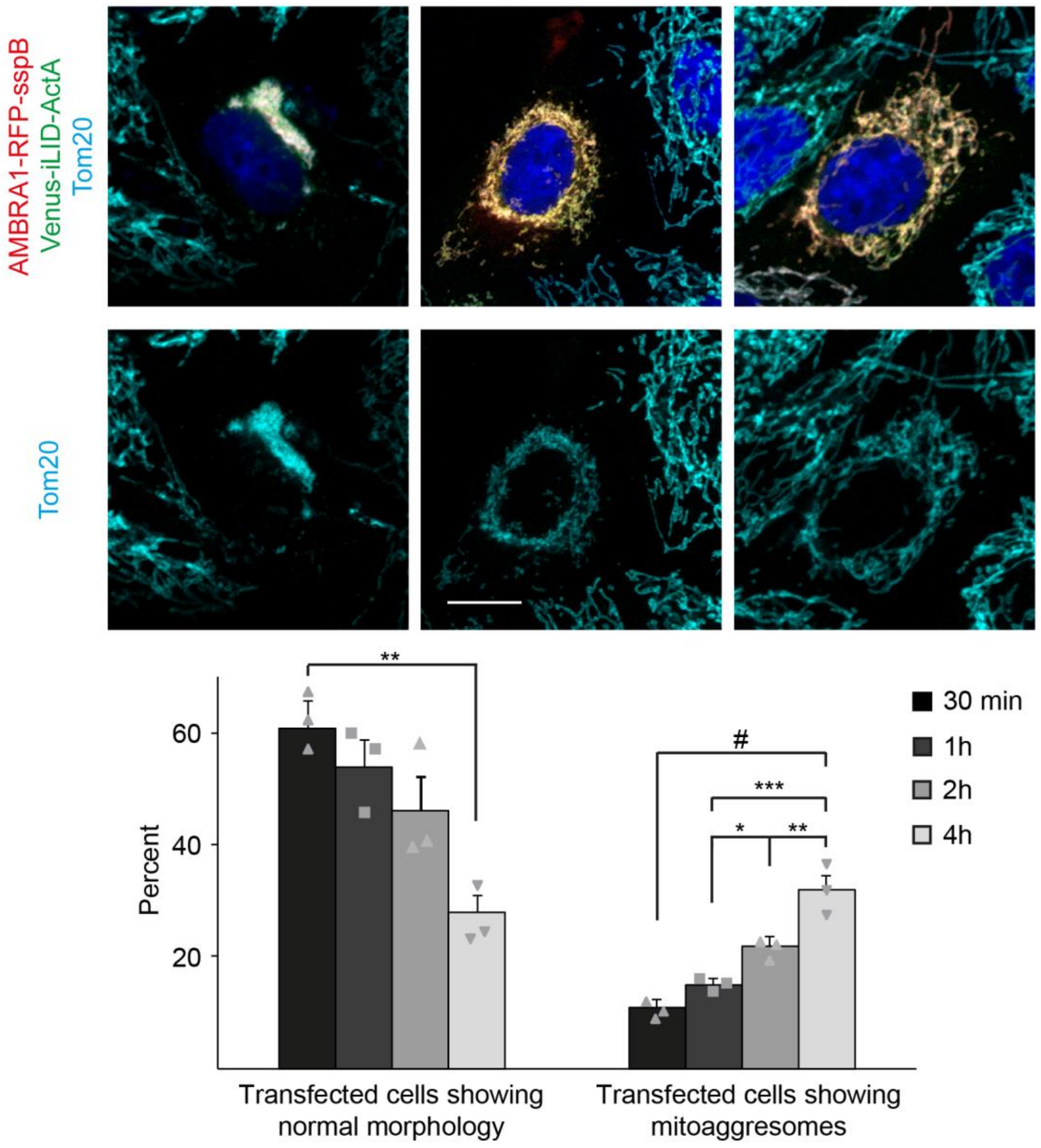

Supplementary Figure 3. *Phenotype classification of Venus-iLID-ActA/AMBRA1- RFP-sspB HeLa cells after 30 min, 1h, 2h and 4h of blue light stimulus.* HeLa cells, manipulated and immunostained as described in Fig. 2a, were illuminated for 0,5h, 1h, 2h, 4h. Focusing on mitochondrial morphology, transfected cells were classified as mitophagic if mitochondria were fragmented and clustered around nuclei (left), uncertain if mitochondria were fragmented but widespread within the cytosol (center) or normal if they were indistinguishable from proximal, not-transfected cells (right). Representative images are shown in the figure. Graphs summarize quantifications in term of percentage of mitophagic and normal cells in 3 independent experiments. A minimum of 120 cells was counted for each experiment. Scale bar: 10 µm. Data shown: Mean: ± S.E.M. Hypothesis test: ANOVA test. *: $p<5x10^{-2}$. **: $p<10^{-2}$. ***: $p<10^{-3}$. #: $p<10^{-4}$. Source data are provided as a Source Data file.

Supplementary Figure 4

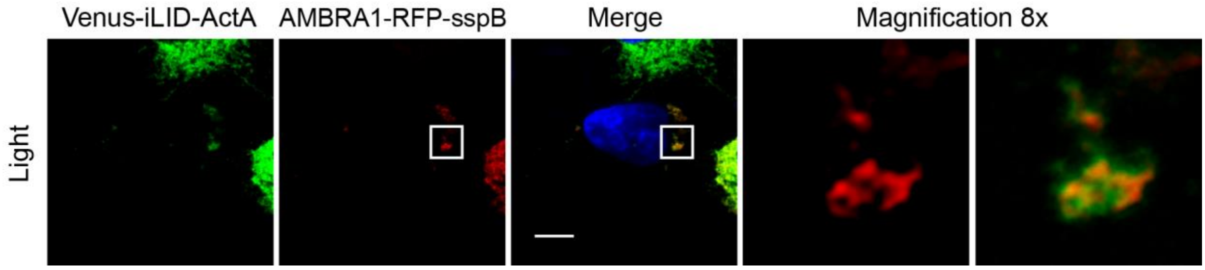

Supplementary Figure 4. *After 24h of pulsed, blue light illumination, Venus-iLID-ActA/AMBRA1-RFP-sspB double positive HeLa cells with only few residual mitochondria could be found.* Representative image of fixed HeLa cells transfected with plasmids encoding Venus-iLID-ActA/AMBRA1-RFP-sspB and illuminated 24h (1s light/1min dark) with sustained mitophagy induction (13,4±3,7% of total transfected cells in 3 independent experiments). Note the round-shaped AMBRA1-RFP-sspB structures surrounding mitochondria in the perinuclear zone, strongly resembling mito-aggresomes. Inset: 8x magnification. Scale bar: 10 μm.

Supplementary Figure 5

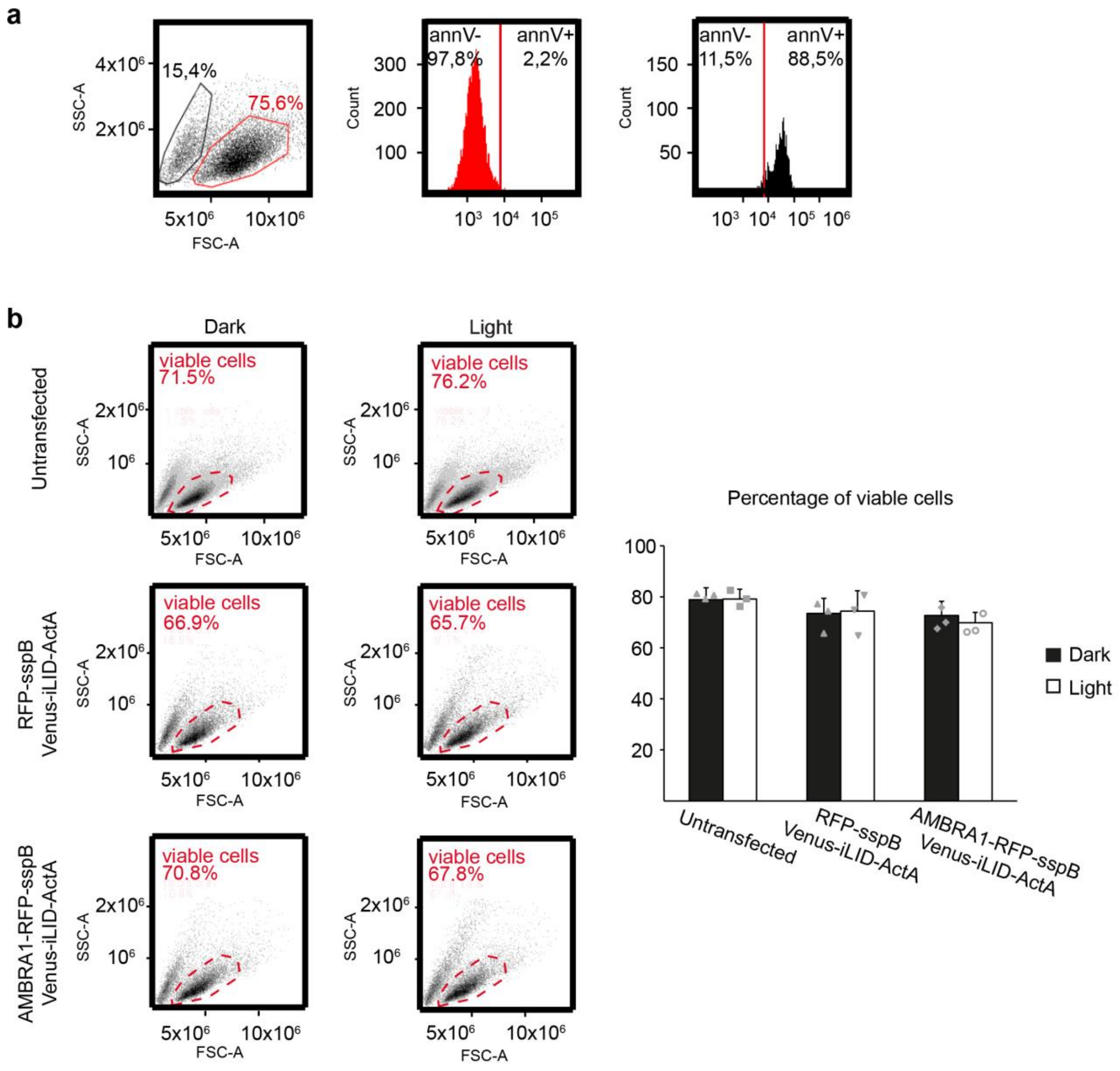

Supplementary Figure 5. *Upon blue light irradiation, cell death in Venus-iLID-ActA/AMBRA1-RFP-sspB HeLa cells is negligible.* (**a**) HeLa cells transfected with an empty pLL 7.0 vector were stained with Annexin-V FITC and analysed by flow cytometry. Apoptotic cells (AnnV+, black graph and circle) were characterized by reduced cell size - low Forward Scattering (FSC) - and enhanced density - high Side Scattering (SSC) - as expected. By contrast, healthy cells showed opposite features (red graph and circle). Thus, we used in downstream applications the FSC/SSC ratio to estimate the percentage of healthy, viable cells. FSC: Forward Scattering. SSC: Side Scattering. (**b**) Co-expressing Venus-iLID-ActA/AMBRA1-RFP-sspB, Venus-iLID-ActA/RFP-sspB or untransfected HeLa cells were analysed by flow cytometry after 48h of dark or pulsed (1s light, 1 min dark) blue light, respectively. The percentage of viable cells was quantified in 3 independent experiments. No statistically significant differences were found. Data shown: Mean ± S.E.M. Hypothesis test: Student’s t test. FSC: Forward Scattering. SSC: Side Scattering. Source data are provided as a Source Data file.

Supplementary Figure 6

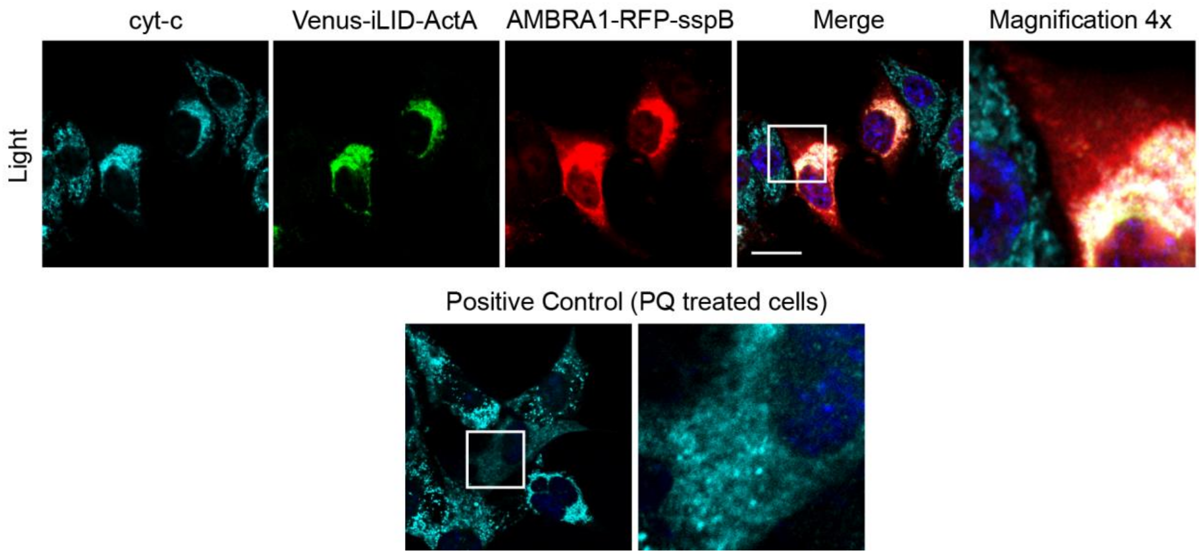

Supplementary Figure 6. *Cytochrome-c is tightly confined within mitochondria upon mito-aggresomes formation***.** Co-expressing Venus-iLID-ActA/AMBRA1-RFP-sspB HeLa cells were illuminated for 24h with pulsed, blue light (1 s light – 1 min dark), fixed and immune-stained for cytochrome-c (cyt-c, cyan). Cells showing mitoaggresomes and a slight AMBRA1-RFP-sspB cytosolic retention were selected in order to analyse cyt-c release from mitochondria (marked by Venus-iLID-ActA). Concomitantly, as a technical positive control for cyt-c release, untransfected HeLa cells were treated with 250 μM PQ, showing cytoplasmic cyt-c staining, as expected. Inset: 4x magnification. Scale bar: 10 μm.

Supplementary Figure 7

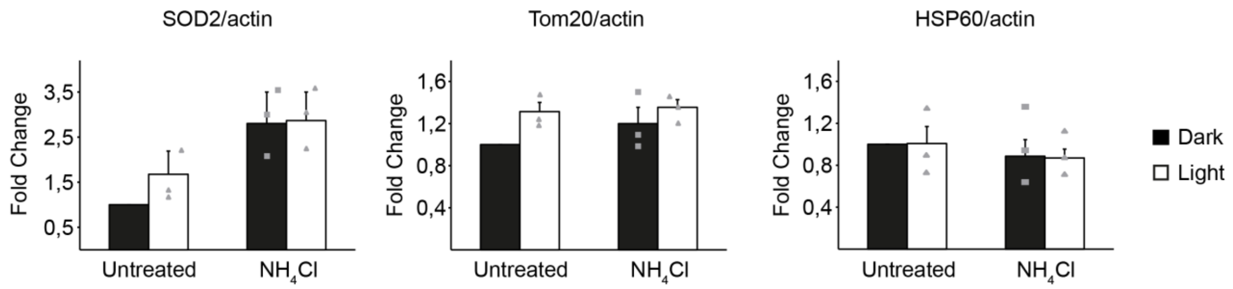

Supplementary Figure 7. *No decrease in mitochondrial markers after 24h of pulsed irradiation in Venus-iLID-ActA/RFP-sspB HeLa cells (negative control)***.** Venus-iLID-ActA/RFP-sspB HeLa cells were handled as described in Fig. 2c. Normalized densitometric analysis of 3 mitochondrial markers over actin ratio was performed in 3 independent experiments and summarized here. A slight but not significant increase was detected for SOD2 and Tom20 markers upon light stimulus. Data shown: Mean ± S.E.M. Hypothesis test: Student's t test. Source data are provided as a Source Data file.

Supplementary Figure 8

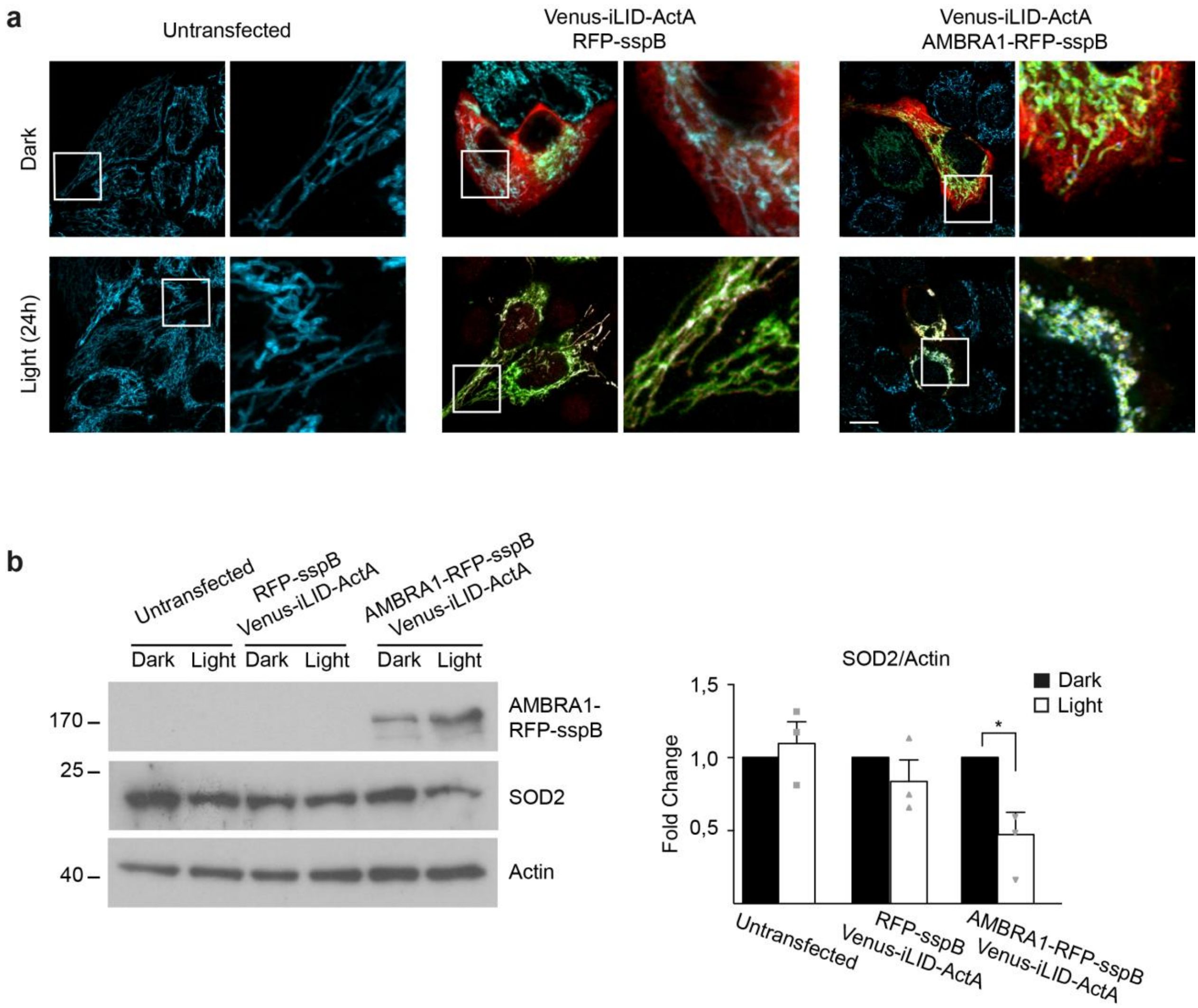

Supplementary Figure 8. *Venus-iLID-ActA/RFP-sspB co-expressing HeLa cells do not show any signs of mitochondrial abnormalities.* (**a**) Untransfected, Venus-iLID-ActA/RFP-sspB and Venus-iLID-ActA/AMBRA1-RFP-sspB co-expressing HeLa cells were irradiated or not for 24h, fixed and immunostained as shown in Fig. 2a to reveal mitochondrial morphology. Cyan: Tom20. Green: Venus-iLID-ActA. Red: RFP-sspB or AMBRA1-RFP-sspB, as indicated. Insets: 4x magnification. Scale bar: 10 µm. (**b**) HeLa cells, overexpressing or not Venus-iLID-ActA/RFP-sspB and Venus-iLID-ActA/AMBRA1-RFP-sspB, were pulse-illuminated for 24h or kept in dark, then lysed and analysed by WB as indicated in the figure. The graph summarizes the densitometric quantification of SOD2 normalized over actin in 3 independent experiments. Data shown: Mean ± S.E.M. Hypothesis test: Student's t test. *: $p<5x10^{-2}$.Source data are provided as a Source Data file.

Supplementary Figure 9

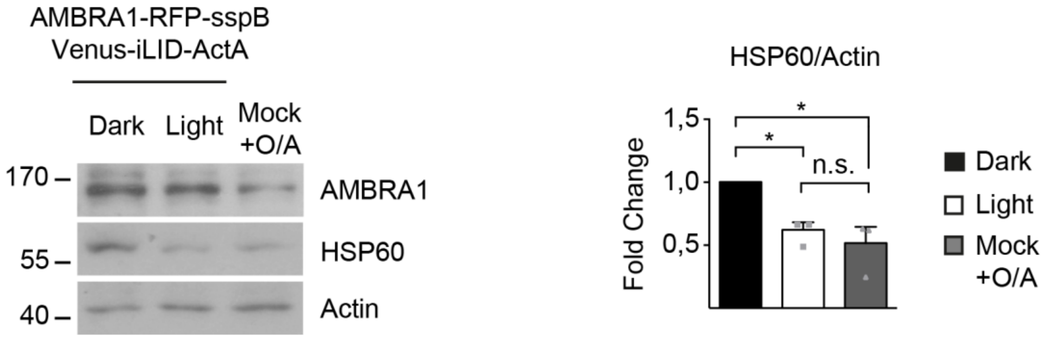

Supplementary Figure 9. *The amplitude of the AMBRA1-RFP-sspB-mediated mitophagy induced in Parkin competent cells is similar to the one achieved with canonical methods.* HEK293T cells were co-transfected with AMBRA1-RFP-sspB and Venus-iLID-ActA and pulsed irradiated or not for 24h. At the same time, mock-transfected cells were treated for 24h with O/A (2.5 μM Oligomycin/0.8 μM Antimycin A) supplemented with the caspase inhibitor QVD to avoid O/A-induced cell death. The graph summarizes the densitometric quantification of the mitochondrial marker HSP60 normalized over actin in 3 independent experiments. Data shown: Mean ± S.E.M. Hypothesis test: ANOVA test. n.s.: not statistically significant. *: $p<5x10^{-2}$. Source data are provided as a Source Data file.

Supplementary Figure 10

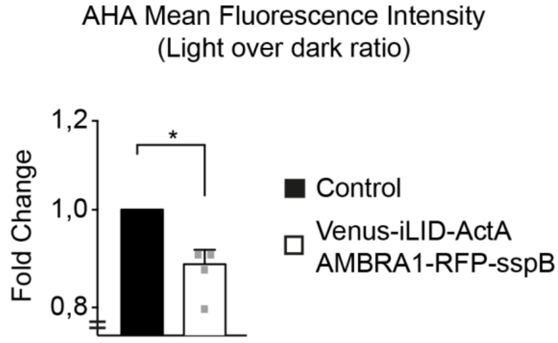

MFI alkyne 647					
Control			Venus-iLID-ActA/AMBRA1-RFP-sspB		
(Ctrl no AHA)	Dark	Light	(Ctrl no AHA)	Dark	Light
(1661)	2377	2307	(2191)	3063	2399

Supplementary Figure 10. AMBRA1-RFP-sspB repositioning to mitochondria induces autophagy. HeLa cells were co-transfected with AMBRA1-RFP-sspB/Venus-iLID-ActA and incubated for 4h with AHA 50µM in a fresh medium without Methionine to stimulate incorporation of AHA. After washing residual AHA with PBS, cells were illuminated (Light) or not (Dark) for 24h. Subsequently, cells were fixed, permeabilized and conjugated with Alexa-647 alkyne to quantify AHA levels by flow cytometry. Positive cells were gated for the YFP signal. As an internal control, untransfected cells naturally present in the same plates were also analysed. The graph summarizes the ratio of Alexa-647 Mean Fluorescence Intensity (MFI) of light over dark resting state in 4 independent experiments; the table in the figure shows the results of a representative experiment. Data shown: Mean ± S.E.M. Hypothesis test: Student's t test. *: $p<5x10^{-2}$. Source data are provided as a Source Data file.

Supplementary Figure 11

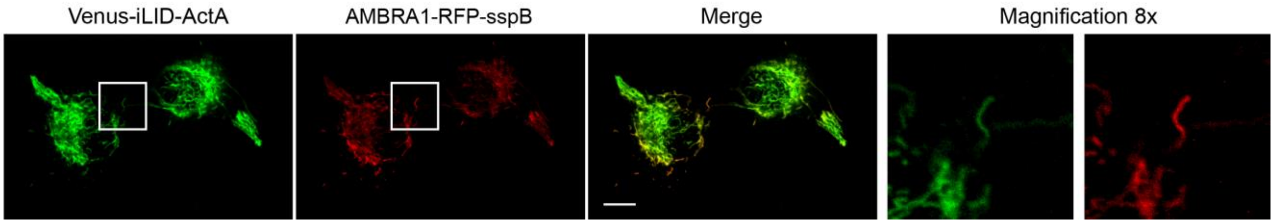

Supplementary Figure 11. *AMBRA1-RFP-sspB is localized to the MOM during live imaging prolonged experiments.* Representative panel showing colocalization of Venus-iLID-ActA/AMBRA1-RFP-sspB after the first light spike of the experiment shown in Fig. 2c and Supplementary Movie 2. Scale bar: 10 μm.

Supplementary Figure 12

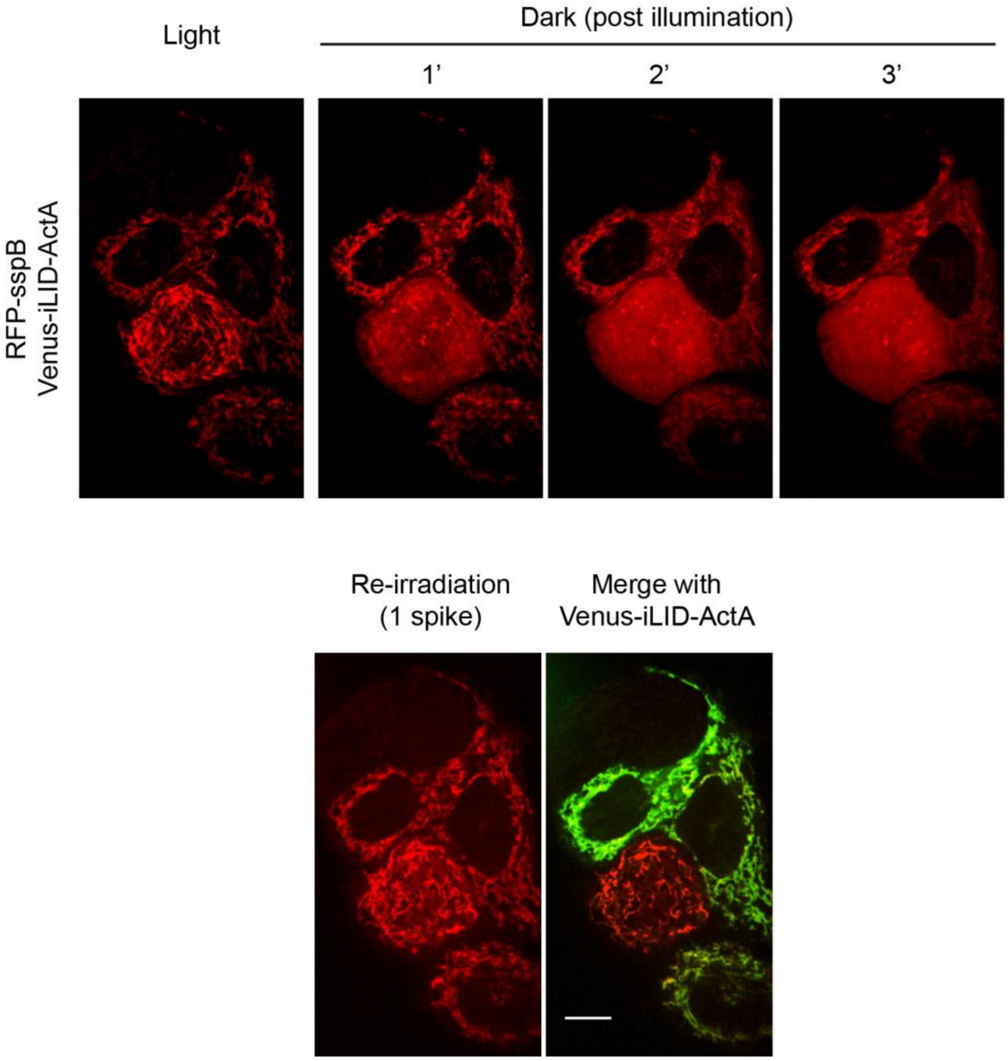

Supplementary Figure 12. *RFP-sspB binding to mitochondria is reversible.* HeLa cells overexpressing Venus-iLID-ActA/RFP-sspB were handled as indicated in Fig. 3a.. Scale bar: 10 µm.

Supplementary Figure 13

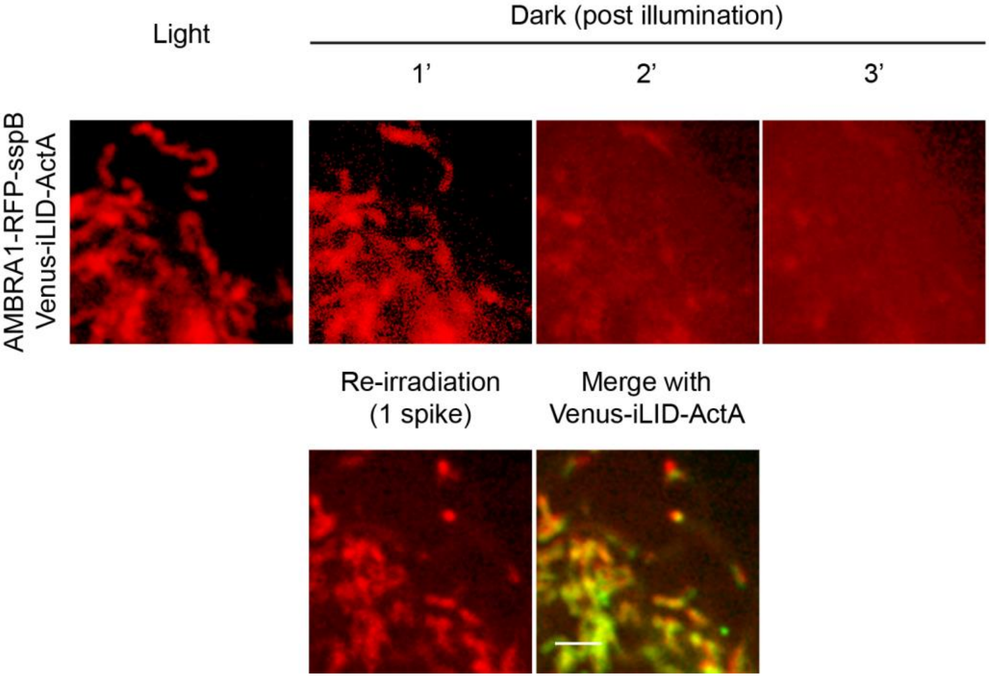

Supplementary Figure 13. *AMBRA1-RFP-sspB binding to mitochondria is reversed with a fast kinetics.* Representative 4x insets of the panel shown in Fig. 3a. Scale bar: 2,5 μm.

Supplementary Figure 14

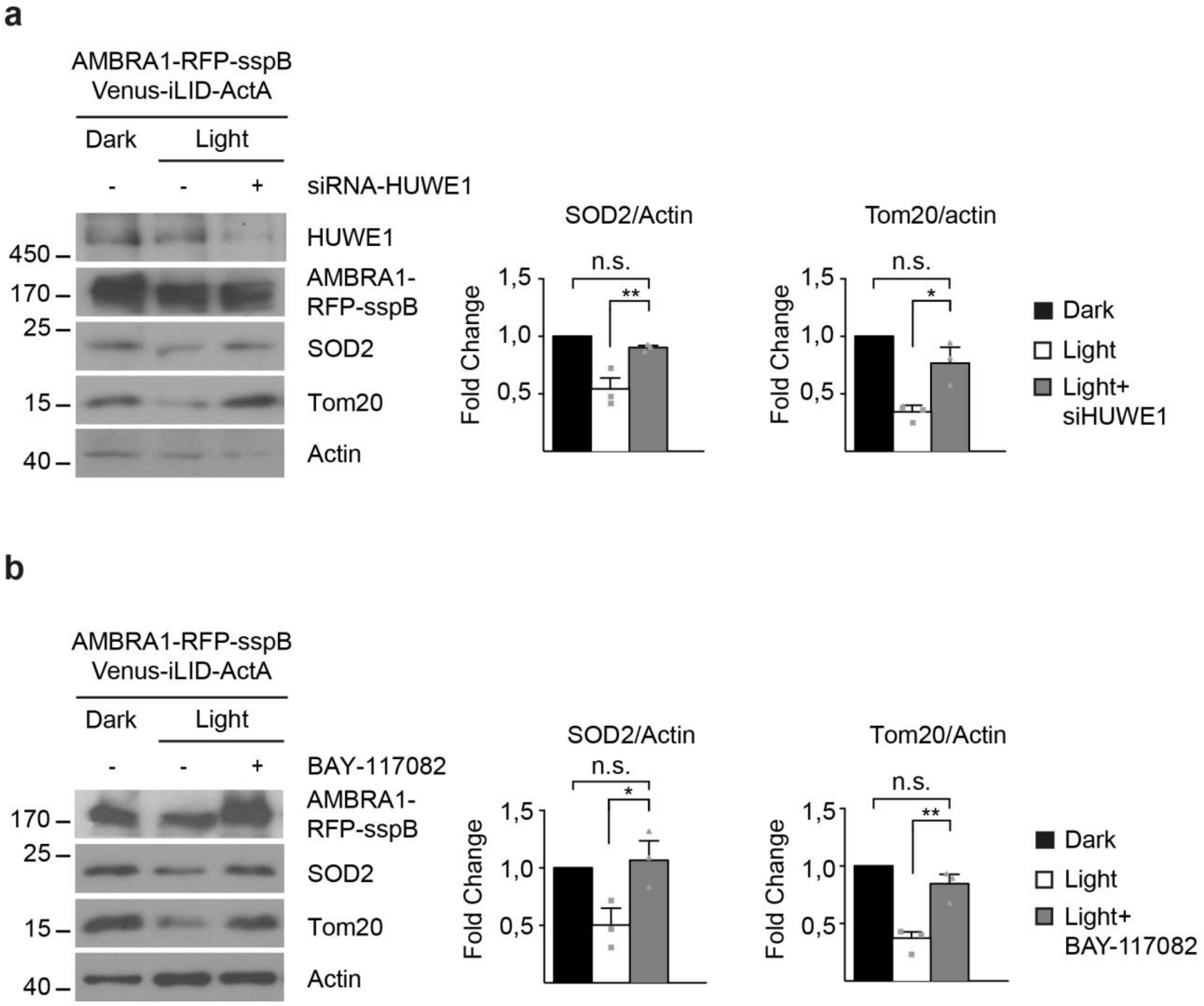

Supplementary Figure 14. *AMBRA1-RFP-sspB-mediated mitophagy is HUWE1- and IKKα-dependent.* (**a**) Venus-iLID-ActA/AMBRA1-RFP-sspB co-expressing HeLa cells were irradiated or not for 24h and interfered for HUWE1 as indicated in the figure. The graphs summarize the densitometric quantification of the mitochondrial markers SOD2 and Tom20 normalized over actin in 3 independent experiments. Data shown: Mean ± S.E.M. Hypothesis test: ANOVA test. n.s.: not statistically significant. *: $p<5x10^{-2}$. **: $p<10^{-2}$. (**b**)Venus-iLID-ActA/AMBRA1-RFP-sspB co-expressing HeLa cells, illuminated or not with pulsed blue light, were treated with the irreversible IKKα inhibitor BAY-117082 and analysed by WB. The graph summarize the densitometric quantification of the mitochondrial markers SOD2 and Tom20 normalized over actin in 3 independent experiments. Data shown: Mean ± S.E.M. Hypothesis test: ANOVA test. n.s.: not statistically significant. *: $p<5x10^{-2}$. **: $p<10^{-2}$. Source data are provided as a Source Data file.

Supplementary Figure 15

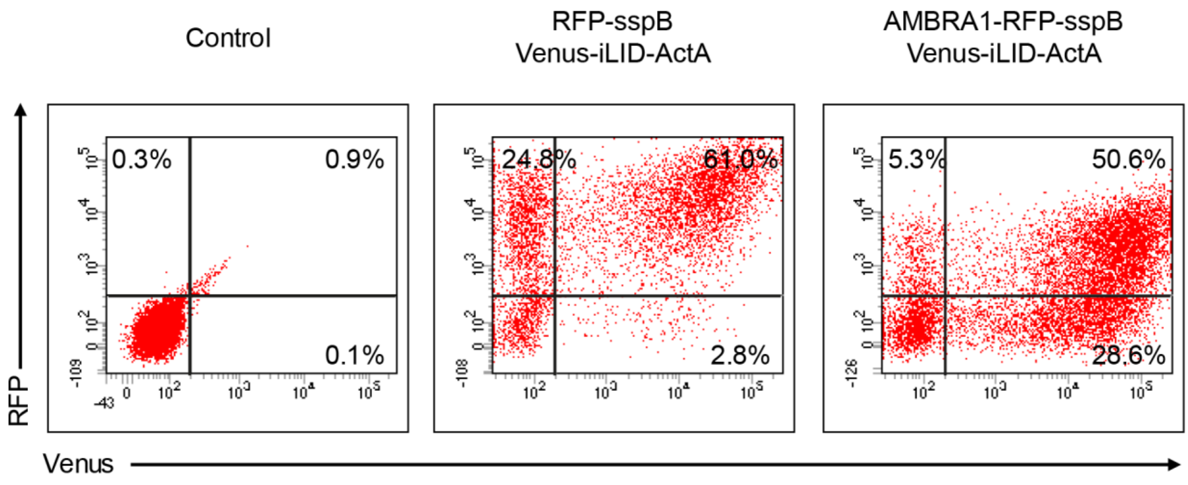

Supplementary Figure 15. *Human T Lymphocytes can be stably infected with retroviruses encoding Venus-iLID-ActA/AMBRA1-RFP-sspB.* Representative flow cytometry analysis of Human T Lymphocytes double infected with retroviral vectors encoding for Venus-iLID-ActA/RFP-sspB (center) and Venus-iLID-ActA/AMBRA1-RFP-sspB (right). The frequencies of double positive cells from 4 different donors were 58,4±7,4% and 42,6±7%, respectively.

Supplementary Figure 16

a

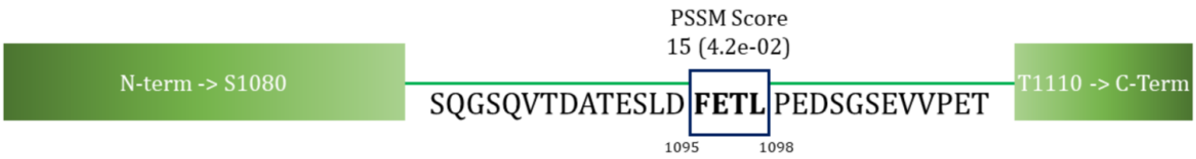

b

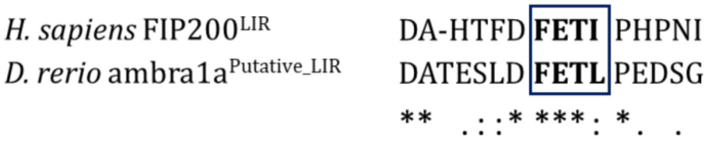

Supplementary Figure 16. *Bioinformatics' analysis of the D. rerio ambra1a protein sequence reveals a putative LIR domain in the C-Term part of the protein.* (**a**) In silico analysis of the zebrafish ambra1a protein sequence through the on-line software iLIR (http://repeat.biol.ucy.ac.cy/iLIR/). The putative LIR with the highest Positive-Specific Scoring Matrix (PSSM) score has been indicated. (**b**) The zebrafish putative LIR has been locally aligned to the experimentally described LIR of the human FIP200 protein (blue box), showing local high similarities.

Supplementary Figure 17

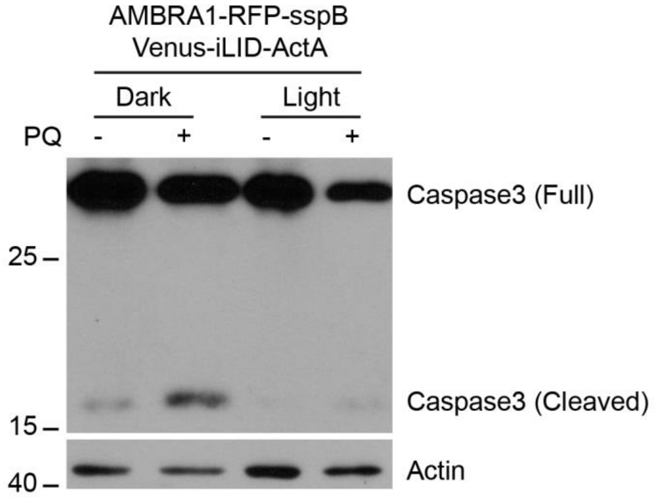

Supplementary Figure 17. *Caspase-3 cleavage analysis confirms the block of apoptosis in Paraquat-treated, Venus-iLID-ActA/AMBRA1-RFP-sspB illuminated ETNA cells.* Representative WB of caspase3 cleavage in ETNA cells manipulated as in Fig. 6b. Actin was used loading control.

Supplementary Figure 18

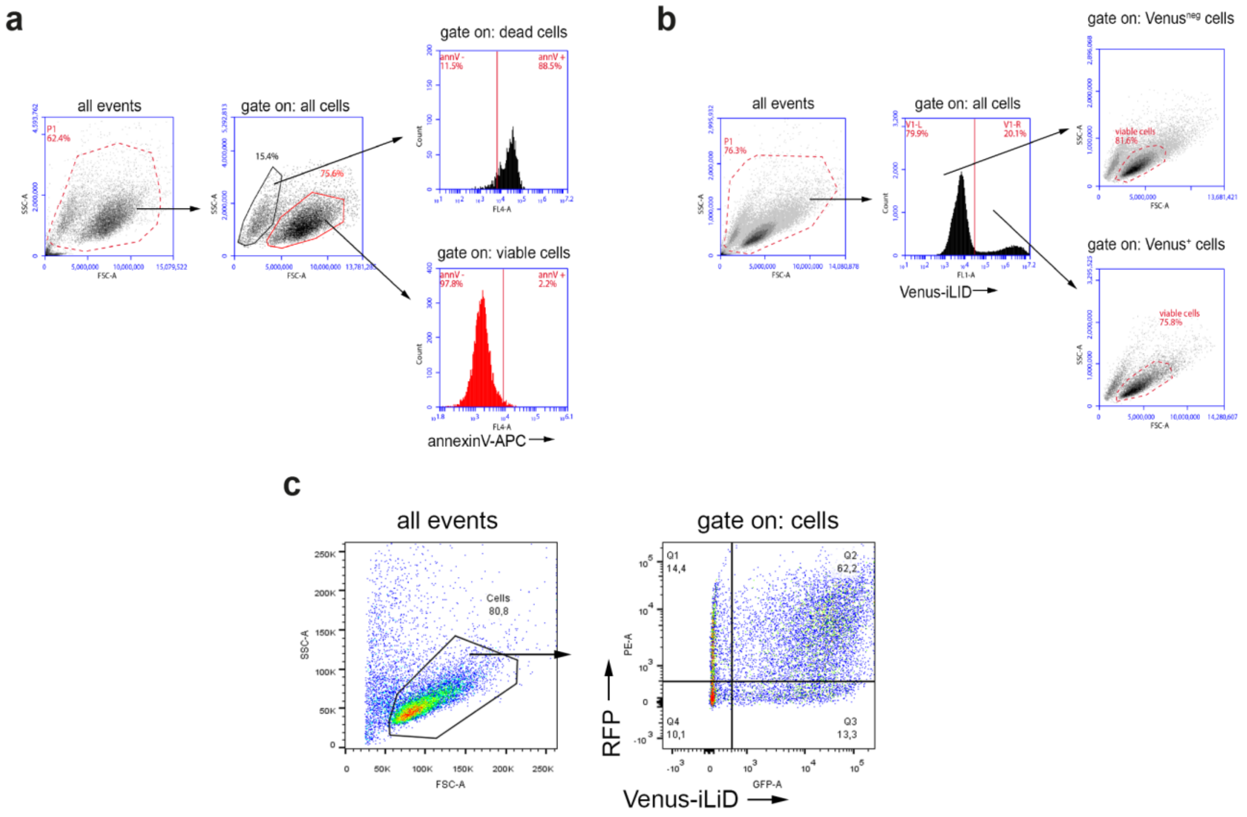

Supplementary Figure 18. *Gating strategies used for flow cytometry analysis.* (**a**) Gating strategy used to analyze cell death in WT HeLa cells, as shown in Supplementary Figure 5a. (**b**) Gating strategy used to distinguish untransfected vs Venus-iLID-ActA/AMBRA1-RFP-sspB (Supplementary Figure 5b and Supplementary Figure 10) or Venus-iLID-ActA/RFP-sspB (Supplementary Figure 5b) co-expressing HeLa cells. (**c**) Gating strategy used to monitor infection efficiency (as reported in Supplementary Figure 15) of human primary T lymphocytes.
