# Supplementary material for: Reversible induction of mitophagy by an optogenetic bimodular system
Source: Nat Commun. 2019 Apr 4;10:1533. doi: 10.1038/s41467-019-09487-1 (PMC6449392; doi:10.1038/s41467-019-09487-1)
Supplement: Supplementary file 3 — Description of Additional Supplementary Files [file 41467_2019_9487_MOESM3_ESM.pdf]

Description of Additional Supplementary Files

File Name: Supplementary Movie 1
Description: AMBRA1-RFP-sspB can be recruited to the MOM with a fast kinetics. Venus-iLID-ActA/AMBRA1-RFP-sspB HeLa cells were live-filmed before (first frame of the video) and during 8 irradiation cycles, consisting of 1 pulse of blue light followed by 35s of dark resting state. Each photogram represents a single irradiation cycle. Green : endogenous fluorescence of the Venus-iLID-ActA fusion protein. Red : endogenous signal of AMBRA1-RFP-sspB

File Name: Supplementary Movie 2
Description: AMBRA1-RFP-sspB-mediated formation of mitoaggresomes. Venus-iLID-ActA/AMBRA1-RFP-sspB HeLa cells were filmed in a timelapse experiment during mito-aggresomes formation in prolonged experiments. The green signal comes from the natural fluorescence of Venus-iLID-ActA and permits to visualize mitochondria in living cells. Each frame corresponds to 1 minute of experiment. Frame rate : 130 sec per frame

File Name: Supplementary Movie 3
Description: AMBRA1-RFP-sspB recruitment to the MOM is reversible. Venus-iLID-ActA/AMBRA1-RFP-sspB HeLa cells were filmed after stimulation of blue light (time zero) and subsequent resting state. The red signal comes from the natural fluorescence of AMBRA1-RFP-sspB. Each frame corresponds to 1 minute of dark resting state.
