# Supplementary material for: Reversible induction of mitophagy by an optogenetic bimodular system
Source: Nat Commun. 2019 Apr 4;10:1533. doi: 10.1038/s41467-019-09487-1 (PMC6449392; doi:10.1038/s41467-019-09487-1)
Supplement: Supplementary file 7 — Reporting Summary Checklist [file 41467_2019_9487_MOESM7_ESM.pdf]

nature research

Corresponding author(s): Francesco Cecconi

Last updated by author(s): Feb 23, 2019

Reporting Summary

Nature Research wishes to improve the reproducibility of the work that we publish. This form provides structure for consistency and transparency in reporting. For further information on Nature Research policies, see Authors & Referees and the Editorial Policy Checklist.

Statistics

For all statistical analyses, confirm that the following items are present in the figure legend, table legend, main text, or Methods section.

n/a	Confirmed	
☐	☒	The exact sample size (n) for each experimental group/condition, given as a discrete number and unit of measurement
☐	☒	A statement on whether measurements were taken from distinct samples or whether the same sample was measured repeatedly
☐	☒	The statistical test(s) used AND whether they are one- or two-sided<br>*Only common tests should be described solely by name; describe more complex techniques in the Methods section.*
☐	☒	A description of all covariates tested
☐	☒	A description of any assumptions or corrections, such as tests of normality and adjustment for multiple comparisons
☐	☒	A full description of the statistical parameters including central tendency (e.g. means) or other basic estimates (e.g. regression coefficient) AND variation (e.g. standard deviation) or associated estimates of uncertainty (e.g. confidence intervals)
☐	☒	For null hypothesis testing, the test statistic (e.g. F, t, r) with confidence intervals, effect sizes, degrees of freedom and P value noted<br>*Give P values as exact values whenever suitable.*
☐	☒	For Bayesian analysis, information on the choice of priors and Markov chain Monte Carlo settings
☐	☒	For hierarchical and complex designs, identification of the appropriate level for tests and full reporting of outcomes
☐	☒	Estimates of effect sizes (e.g. Cohen's d, Pearson's r), indicating how they were calculated

Our web collection on statistics for biologists contains articles on many of the points above.

Software and code

Policy information about availability of computer code

Data collection	No codes used in this study
Data analysis	No codes used in this study

For manuscripts utilizing custom algorithms or software that are central to the research but not yet described in published literature, software must be made available to editors/reviewers. We strongly encourage code deposition in a community repository (e.g. GitHub). See the Nature Research guidelines for submitting code & software for further information.

Data

Policy information about availability of data

All manuscripts must include a data availability statement. This statement should provide the following information, where applicable:

- Accession codes, unique identifiers, or web links for publicly available datasets
- A list of figures that have associated raw data
- A description of any restrictions on data availability

The authors declare that all data supporting the findings of this study are available within the paper and its supplementary information files. No datasets were generated in this study. The source data underlying Figs 1b-d, 2b–c, 3a-b, 4a-b, 5b-c, 6b-c and Supplementary Figures 1, 3, 5, 7, 8b, 9, 10, 14, 17 are provided as a Source Data file

Field-specific reporting

Please select the one below that is the best fit for your research. If you are not sure, read the appropriate sections before making your selection.

☒ Life sciences ☐ Behavioural & social sciences ☐ Ecological, evolutionary & environmental sciences

For a reference copy of the document with all sections, see nature.com/documents/nr-reporting-summary-flat.pdf

Life sciences study design

All studies must disclose on these points even when the disclosure is negative.

Sample size	No statistical methods were used to predetermine sample size.
Data exclusions	No data were excluded from analysis.
Replication	All experiments have been performed at least three times independently. All attempts at replication were successful.
Randomization	Allocation was random in all experiments.
Blinding	Immunofluorescence images were acquired by a blind observer. Western blot and flow cytometry data did not required any blind operator.

Reporting for specific materials, systems and methods

We require information from authors about some types of materials, experimental systems and methods used in many studies. Here, indicate whether each material, system or method listed is relevant to your study. If you are not sure if a list item applies to your research, read the appropriate section before selecting a response.

Materials & experimental systems

n/a	Involved in the study	
☐	☒	Antibodies
☐	☒	Eukaryotic cell lines
☒	☐	Palaeontology
☐	☒	Animals and other organisms
☐	☒	Human research participants
☒	☐	Clinical data

Methods

n/a	Involved in the study	
☒	☐	ChIP-seq
☐	☒	Flow cytometry
☒	☐	MRI-based neuroimaging

Antibodies

Antibodies used	Rabbit α-Actin (Cell Signalling, 4967)<br>Mouse α-AMBRA1 (Santa Cruz Biotechnology, sc-398204)<br>Rabbit α-Casp3 (Cell Signalling, 9662)<br>Mouse α-GAPDH (Chemicon International, now MerckMillipore Mab374)<br>Rabbit α-HSP60 (Santa Cruz Biotechnology, sc-13966)<br>Rabbit α-PARP (Cell Signalling, 9542)<br>Rabbit α-SOD2 (Enzo LifeScience, ADI-SOD-110)<br>Mouse α-Tom20 (Santa Cruz Biotechnology, sc-17764)<br>Mouse α-Tubulin (Sigma-Aldrich, T6199)<br>rabbit α-Tom20 (Santa Cruz Biotechnology, sc-11415)<br>anti-CD3 (OKT3, eBioscience, 14-0037-82)<br>anti-CD28 (BD Biosciences, 555726)<br>rabbit a-HUWE1 (Bethyl, A300-486A)
Validation	All antibodies are validated by manufacturers. In addition, all mitochondrial antibodies have been validated by us through colocalization with the mitochondrial reporter Venus-iLID-ActA and/or biochemically through mitochondria/cytosol purification (see Fig. 1b). HUWE1 antibody was also validated by us through siRNA (see Supplementary Figure 14). Finally, PARP and casp3 antibodies were validated in our experiments by the appearance of a cleaved PARP/casp3 band when apoptosis was induced (see Fig. 6c and Supplementary Figure 17).

Eukaryotic cell lines

Policy information about cell lines

Cell line source(s)	HeLa, HEK293T, ETNA, 293T/17
Authentication	HeLa (catalogue number 93021013) and HEK cells (ECACC 12022001) have been purchased from Sigma. ETNA cells have been immortalized in our laboratory from embryonic striatum primordia murine E14.5 neurons, as described in Cozzolino et al., 2004 (included in the reference list). The 293T/17 packaging cell line (ATCC® CRL-11268) was authenticated by short tandem repeat analysis by an external certified company (Eurofins Genomics). All cell lines were routinely tested for mycoplasma contamination
Mycoplasma contamination	All cell lines tested negative for mycoplasma contamination
Commonly misidentified lines (See ICLAC register)	None

Animals and other organisms

Policy information about studies involving animals; ARRIVE guidelines recommended for reporting animal research

Laboratory animals	Zebrafish (D. rerio) of wild type strain were maintained at 28.5°C in a Tecniplast acquarium system. Embryos were obtained by natural breeding and raised in Petri dishes containing Fish Water solution at 28.5°C with a photoperiod of 14 h light/10 h dark. Developmental stages of embryos were determined according to the time after fertilization and morphological criteria.
Wild animals	This study did not involve wild animals
Field-collected samples	This study did not involve samples collected from the field
Ethics oversight	All husbandry and experimental procedures complied with European Legislation for the Protection of Animals used for Scientific Purposes (Directive 2010/63/EU). EU does not require specific approval for experimental procedures done with zebrafish embryos.

Note that full information on the approval of the study protocol must also be provided in the manuscript.

Human research participants

Policy information about studies involving human research participants

Population characteristics	Peripheral blood mononuclear cells (PBMC) were isolated from buffy coats obtained from adult volounteeers (healthy donors) from Bambino Gesù Children's Hospital (OPBG) in Rome, Italy, who signed a written informed consent, in accordance with rules set by the Institutional Review Board of OPBG (Approval of Ethical Committee N°969/2015 prot. N° 669LB)
Recruitment	Human adult blood donor volounteers
Ethics oversight	Bambino Gesù Children Hospital

Note that full information on the approval of the study protocol must also be provided in the manuscript.

Flow Cytometry

Plots

Confirm that:

☒ The axis labels state the marker and fluorochrome used (e.g. CD4-FITC).

☒ The axis scales are clearly visible. Include numbers along axes only for bottom left plot of group (a 'group' is an analysis of identical markers).

☒ All plots are contour plots with outliers or pseudocolor plots.

☒ A numerical value for number of cells or percentage (with statistics) is provided.

Methodology

Sample preparation	HeLa cells have been trypsinazed and harvested, washed twice with PBS and incubated for 15min with AnnexinV-FITC antibody (AnnexinV Apoptosis Detection Kit) then washed and read. For pulse-chase experiments, HeLa cells have been fixed and stained with Cell Reaction Buffer Kit (Invitrogen) using Alkyne-Alexa647 conjugation with pre-incubated AHA reagent (Invitrogen). Peripheral blood mononuclear cells (PBMC) were isolated from buffy coats, using Lymphocytes separation medium (Eurobio, France). T lymphocytes were activated with immobilized OKT3 (1 µg/ml, e-Bioscience, California, USA) and anti-CD28 (1 µg/ml, BD Biosciences, Europe) monoclonal antibodies in the presence of recombinant human interleukin-7 (IL7, 10 ng/ml; R&D, USA) and 15 (IL15, 5 ng/ml; R&D)41. Activated T cells were transduced on day 3 in 24-well plates pre-coated with recombinant human RetroNectin (Takara-Bio, Japan) using a specific retroviral supernatant. On day 5 after transduction, T cells were expanded in

medium containing 45% RPMI1640 and 45% Click's medium (Sigma-Aldrich) supplemented with 10% FBS and 2 mM Glutamax and replenished twice a week. Expression of the CD3 cell surface molecule, the green and red fluorescence proteins were determined by flow-cytometry using standard methodology. For each sample, we analyzed a minimum of 20,000 events.

Instrument	Accuri C6; FACS Celesta; BD LSRFortessa X-20
Software	FACSDiva; Accuri C6 software
Cell population abundance	No sorting procedures used.
Gating strategy	AnnexinV: HeLa cells identified with FSC/SSC<br>Pulse-chase and viability analysis: HeLa cells identified with FSC/SSC have been further subdivided into non trasfected YFP-negative cells and transfected YFP-positive cells (on which Alexa-647 median fluorescence intensity was measured).<br>Human T cells: identified with FSC/SSC. Then cells have been analysed for green (Venus-YFP) and red (Ambra-RFP) fluorescences.<br>A detailed Supplementary Figure (supplementary figure 18) has been provided showing gating strategy

☒ Tick this box to confirm that a figure exemplifying the gating strategy is provided in the Supplementary Information.
